# Repellent activities of dichloromethane extract of *Allium sativum* (garlic) (Liliaceae) against *Hyalomma rufipes* (Acari)

**DOI:** 10.4102/jsava.v87i1.1356

**Published:** 2016-12-02

**Authors:** Felix Nchu, Solomon R. Magano, Jacobus N. Eloff

**Affiliations:** 1 Department of Horticultural Science, Cape Peninsula University of Technology, South Africa; 2 Department of Life and Consumer Sciences, University of South Africa, Florida Campus, South Africa; 3 Department of Paraclinical Sciences, University of Pretoria, Onderstepoort, South Africa

## Abstract

Dichloromethane (DCM) extract of garlic (*Allium sativum* Linn.) bulbs was assessed for its repellent effect against the hard tick, *Hyalomma rufipes* (Acari: Ixodidae) using two tick behavioural bioassays; Type A and Type B repellency bioassays, under laboratory conditions. These bioassays exploit the questing behaviour of *H. rufipes*, a tick that in nature displays ambush strategy, seeking its host by climbing up on vegetation and attaching to a passing host. One hundred microlitres (100 µL) of the test solution containing DCM extract of garlic bulbs and DCM at concentrations of 0.35%, 0.7% or 1.4% w/v were evaluated. DCM only was used for control. Tick repellency increased significantly (*R*^2^ = 0.98) with increasing concentration (40.03% – 86.96%) yielding an EC_50_ of 0.45% w/v in Type B repellency bioassay. At concentration of 1.4% w/v, the DCM extract of garlic bulbs produced high repellency index of 87% (male ticks) and 87.5% (female ticks) in the Type A repellency bioassay. Only 4% avoidance of male ticks or female ticks was recorded in the Type B repellency bioassay. In the corresponding controls, the mean numbers of non-repelled male or female ticks were 80% and 41 males or 38 females of 50 ticks in the Type A and Type B repellency bioassays, respectively. The variations in the results could be attributed to the difference in tick repellent behaviours that were assessed by the two repellency bioassays; the Type A repellency bioassay assessed repellent effect of garlic extracts without discriminating between deterrence and avoidance whereas the Type B repellency bioassay only assessed avoidance response. Generally, DCM extract of garlic was repellent against *H. rufipes*, albeit weak tick repellency was obtained in the Type B repellency bioassay. Furthermore, this study established that the tick repellent activity of garlic extracts is predominantly by deterrence.

## Introduction

Tick bites and tick-borne diseases resulting from encounters with ticks are widespread; for example, in South Africa, there are reports of ixodids such as *Hyalomma* and *Amblyomma* feeding on humans (Estrada-Pẽna & Jongejan 1999; Horak *et al*. 2002). While synthetic repellents are being used extensively for reduction of tick infestations on hosts, there is a growing awareness of the risks associated with the rampant use of these substances (Bissinger & Roe 2010). Consequently, there is mounting interest in the use of alternative and environmentally friendly arthropod pest control agents such as plant-based repellents (Kaaya 2003; Pålsson & Jaenson 1999). *Allium sativum* L. is attracting interest from researchers as a potential source of tick repellent, especially following a report that the consumption of garlic protected soldiers exposed to ticks from tick bites under field conditions (Stjernberg & Berglund 2000). Also, there are many anecdotes and ethnobotanical claims suggesting that garlic extracts repel pest of arthropod origin (Karunamoorthi & Hailu 2014). Nevertheless, very few studies have scientifically validated these claims and most of the scientific investigations involving garlic repellent activities have been directed towards insects (Birrenkott *et al*. 2000; Kianmatee & Ranamukhaarachchi 2007; Sritabutra *et al*. 2011).

Many reasons have been put forward to explain this surprising lack of progress towards development of plant-based tick repellent products. There are considerable knowledge gaps on the repellent activities of botanicals against ticks, for example, the study of Stjernberg and Berglund (2000) only focused on the repellent effect of oral garlic on unspecified tick species. Few *in vitro* repellent bioassays have been developed and there is generally a lack of standard procedures to test for tick repellency. Moreover, the few *in vitro* repellency bioassays that have been developed so far have shown some limitations, for example, different repellency bioassays produce contrasting results even when using the same tick species (Carroll *et al*. 2004). At least two reasons could be postulated for the varied tick responses to the currently developed repellency bioassays. Firstly, natural behaviour of ticks is sometimes not sufficiently taken into account during experimental design. Secondly, differentiating between the repellents and deterrents is not always easy as there may be continuous input of olfactory stimuli during contact between the pest and extract (Koshier & Sedy 2003). Bioassays that sufficiently take tick behaviour into account and are able to discriminate between deterrent stimulus and repellent stimulus are therefore necessary (Liu & Ho 1999). It is important to specify whether a repellent is a contact repellent (deterrent) or non-contact repellent (elicits avoidance response in ticks) to minimise confusion, because a contact repellent (deterrent) may not necessarily be a non-contact repellent. Bissinger and Roe (2010) argued that the relative importance of olfaction versus tactile chemoreception in repellency is currently under-appreciated. For the purpose of this study, a tick repellent is defined as a substance whose stimulus elicits an avoidance response in ticks and/or prevents ticks from settling on a vantage position for a specified time period. In the present study, two types of climbing repellency bioassays developed in our laboratory were used to screen for the repellent effects of extracts of *A. sativum* on adults of *Hyalomma rufipes* as well as to establish whether the garlic extract is a contact or non-contact repellent.

## Materials and methods

### Ticks

Host-seeking (3–4 weeks old) *Hyalomma rufipes* adult ticks used in this study were obtained from laboratory colonies maintained on New Zealand white rabbits in the Department of Biology at the Sefako Makgato Health Sciences University (formerly known as the University of Limpopo). These ticks were maintained in glass humidity chambers at 25 ºC ± 1 ºC, 75% ± 5% RH and natural day or night regimen.

### Extraction of plant material

Fresh garlic (*A. sativum*) bulbs used in this study were obtained from a vegetable store in Pretoria North. Dichloromehtane (DCM) extracts of *A. sativum* were prepared by simply introducing 10 g of crushed fresh garlic bulb into 20 mL of DCM for 90 min. The same procedure was repeated twice. The crude mixture of the plant material and the solvent was allowed to stand for 90 min following which the supernatant was filtered out with the aid of Whatman Number 1 filter paper. The DCM was evaporated completely using a fan at room temperature for 4 h. The residues obtained were re-dissolved in varied volumes of DCM in order to obtain the following concentrations: 0.35%, 0.7% and 1.4% w/v. These concentrations of DCM extracts of garlic bulb were assessed in the Types A and B repellency bioassays described below.

### Repellency bioassays

#### Type A repellency bioassay

The Type A tick climbing repellency bioassay described in this study or its variations have been used successfully by workers in our laboratory to evaluate tick repellency of essential oils and crude extracts of plants and DEET (N,N-diethyl-methyl-m-toluamide) (Mkolo & Magano 2007; Nchu, Magano & Eloff 2012; Zorloni, Penzhorn & Eloff 2010). This bioassay is based on the climbing behaviour of host-seeking ticks. Two glass rods of a similar length were each vertically and firmly fixed on a polyesteryn platform (*L* = 5 cm, *W* = 5 cm, *H* = 3.5 cm). Twenty-one centimetres of each glass rod was exposed above its platform. The two platforms with inserted glass rods were fixed separately on the inside of a plastic container (*L* = 35 cm, *W* = 24 cm and *H* = 8 cm) (Nchu *et al*. 2012). Water was added to the container in such a way that it completely surrounded each of the platforms and almost reached the height of each of the platforms. DCM extracts of garlic, 100 µL of the test solution was released on the test filter paper strip (Whatman No. 1) (2.5 cm × 5 cm). The control filter paper strip was of the same kind and size (Nchu *et al*. 2012). However, only DCM was released on the control filter paper strip. After air-drying the filter paper strips (by evaporating the solvent), the test filter paper was used to cover the last 5 cm of the test glass rod while the control filter paper was used to cover the similar region of the control glass rod. Two more filter paper strips (neutral filter paper strips) of the same size (2.5 cm × 1.5 cm) were each fixed below the test and control filter papers on the glass rods so that the two adjacent edges of the two filter papers touched each other. Surgical gloves were used to handle the glass rods and filter papers prior and during the experimentation period. Ten ticks of the same sex were released on each platform and were allowed to climb the glass rods. Each treatment was replicated five times.

The positions of ticks were recorded after an hour following the start of the experiment. Ticks that were found on upper filter paper were considered not repelled. Those on the bottom filter paper, naked part of the glass rod and on the platform were considered repelled. Ticks that moved into water were dried and replaced or re-introduced onto the platform (Nchu *et al*. 2012). The repellent effect was calculated as percentage repellency according to the formula: Percentage repellency = 100 – (Mean no. of ticks on test/mean no. of ticks on control) × 100 (Jantan & Zaki 1998).

#### Type B repellency bioassay (avoidance bioassay)

Basically, this bioassay is very similar to bioassay A except for slight modifications. The previous bioassays showed limitations, since discrimination between the effects of the stimulus that produces avoidance in ticks and deterrent stimulus could not be established. This behavioural bioassay provides clarity on the nature (volatiles or fixed oils) of the plant responsible for tick repellency. Only the highest concentration, that is, 1.4% w/v, for DCM extract of garlic was used for treatments on the filter paper strips. DCM only was used for control. Adult ticks were placed one after the other on each platform and allowed to climb the glass rod. A total of 50 ticks (five replications of ten ticks each in both control and test) were used. This was done to minimise the amount of volatiles lost to the surrounds during the duration of the experiment. However, the outcome for each tick was considered as independent. Since adults of *H. rufipes* tend to have a strong inclination to climb to the topmost part of glass rods in tick climbing repellency bioassays, ticks that climbed and stopped at the bottom filter paper for 45 seconds without moving onto the top filter paper of the treatment glass rods were considered to be avoiding the extract and removed. Other researchers, for example, Lerdthusnee *et al*. (2003) and Carroll *et al*. (2004) used different time periods (5 s and 3.5 min respectively) to evaluate candidate arthropod repellents. The average laboratory conditions during the study were 25 °C ± 5 °C and 50% ± 10% RH. Experiments were conducted under a natural day/night regimen. However, repellency bioassays were performed during daytime only; light sources were the window and ceiling fluorescent lamp.

### Data analysis

For the Type A repellency bioassay, data are presented as percentage repellency and proportion of ticks repelled according to sex. Data were transformed to arc sin square root of the proportion of ticks repelled prior to subjecting it to one-way independent Analysis of variance (ANOVA) (Hammer *et al*. 2001). The repellent responses of males and females for each concentration were pooled for one-way ANOVA, because no significant difference (*p* < 0.05) was found between males and females at all concentrations of DCM extract of *A. sativum*. However, mean ± SE of untransformed data are reported. In the Type B repellency bioassay, comparison between control and test results for proportion repelled in both male and female trials were done using Fisher’s test. The effective concentration needed to repel 50% ticks (EC_50_) was determined by probit statistics (Finney 1971) using BioStat V5 (AnalystSoft 2014). Statistical level of significance was kept at *p* < 0.05.

## Results

### Tick repellency

**FIGURE 1:**
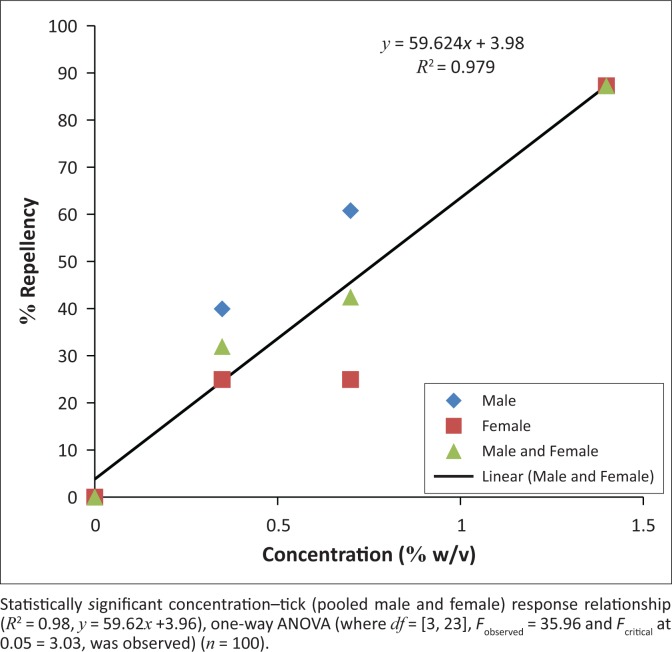
Relationship between % repellency and concentrations of dichloromethane extract of *Allium sativum* on adults of *Hyalomma rufipes* in the Type A repellency bioassay.

In the Type A repellency bioassay, fewer ticks were observed on test filter papers than on control filter papers at all concentrations of DCM extract of garlic (Figure 1). A noteworthy observation is that no significant difference was found between male and female responses at all concentrations of DCM extract of *A. sativum*. As a result, male and female data were pooled prior to statistical analysis. There was a significant (*R*^2^ = 0.98, *y* = 59.62*x* +3.96) dose – tick repellency response relationship and the tick repellencies induced by garlic ranged from 40.03% to 86.96% from the lowest (0.35% w/v) to the highest (1.4% w/v) garlic extract concentrations (Figure 1), producing an EC_50_ of 0.45% w/v (CI = 0.29% – 0.58% w/v) (Table 1). During the experiment, ticks (both test and control ticks) were found to prefer questing on filter papers to naked regions of the glass rods or resting on the platform. Test and control ticks also displayed aggregation behaviour (ticks congregate) on filter paper strips (neutral and control filter paper strips, respectively) (Figure 1).

**TABLE 1:** Effective dose needed to repel 50% of ticks in the Type A repellency bioassay following exposure of adults *Hyalomma rufipes* ticks to dichloromethane extracts of *Allium sativum* at concentrations of 0.35%, 0.7% and 1.4% w/v.

Effective dose needed to repel 50% of ticks (ED_50_)	Lower confidence limit (CI)	Upper confidence limit (CI)
0.45% w/v	0.29% w/v	0.58% w/v

At concentration of 1.4% w/v, the DCM extract of garlic bulbs produced a high repellency index of 87% (male ticks) and 87.5% (female ticks) in the Type A repellency bioassay, whereas only 4% avoidance of male ticks or female ticks was recorded in the Type B repellency bioassay (Table 2). In the corresponding controls, the mean numbers of non-repelled ticks were 8 of 10 male or female ticks and 41 male ticks or 38 female ticks of 50 ticks in the Type A and Type B repellency bioassays, respectively (Figure 1 and Table 2).

**TABLE 2:** Summary of the results obtained in the Type B repellency bioassay using dichloromethane extracts of *Allium sativum* against males and females of *Hyalomma rufipes*.

DCM extract of *Allium sativum* – Conc. (% w/v)	Number of ticks used		Number of ticks avoiding extract		% avoidance for a maximum of 45 seconds	
	M	F	M	F	M	F
1.4	50	50	2	2	4	4
0	50	50	2	2	4	4

DCM, Dichloromethane; Conc., concentration; M, male; F, female.

No statistical significance (*p* > 0.05) in the number of ticks repelled between control and test.

## Ethical approval

This study was approved by the Animal Ethics Committee, University of Limpopo, Medunsa campus (now Sefako Makgato Health Sciences University) and rabbits used were treated humanely.

## Discussion

Generally, DCM extract of garlic showed positive repellent effects on ticks at all concentrations tested in the Types A and B repellency bioassays with a dose-dependent response in the Type A bioassay and an EC_50_ of 0.45% w/v. Previously, Mkolo and Magano (2007), using the same bioassay as the current Type A repellency bioassay to assess repellency of DEET on *H. m. rufipes* (presently known as *H. rufipes*) adults, obtained an EC_50_ of 4.7% v/v. The current results corroborate previous reports on the repellent activities of garlic (Stjernberg & Berglund 2000). Garlic contains volatile compounds that are mainly non-polar compounds and the bioactivity or repellency induced by these compounds could be related to higher volatility and the presence of functional groups that are capable of reacting with sensory receptors involved (Gaddaguti *et al*. in press). The olfactory sensilla and contact chemosensilla are responsible for the perception of volatile and non-volatile stimuli, respectively (Bissinger & Roe 2013).

Discrepancies in the results obtained in the two bioassays used in this study can also be associated with the differences in the tick bioassays and the corresponding tick behaviours that were assessed. At times, bioassays that differ in seemingly minor ways can yield surprisingly different results (Carroll *et al*. 2004). Nevertheless, these climbing bioassays seem to be widely applicable and suitable for hard ticks (Carroll *et al*. 2004). Moreover, it should be considered that the response of ticks to repellents varies from one tick species to the other as well as from one stage of the tick life-cycle to the other. *Ixodes scapularis* and *Amblyomma americanum* responded differently to the same concentrations of N,N-diethyl-methyl-benzamide (DEET) (Carroll *et al*. 2004). According to Dautel *et al*. (1999), N,N-diethyl-m-toluamide (DEET) elicited avoidance reaction of *Ixodes ricinus* nymphs *in vitro* but McMahon, Kröber and Guerin (2003) found no avoidance response to N,N-diethyl-methyl-benzamide (DEET) by *I. ricinus* adults. Therefore, to avoid conflicting results it is important to always specify the species used and the developmental stage of the ticks.

The use of the word repellent may in some instances lead to confusion. It is important to clearly explain or define what is meant by a repellent and clearly distinguish contact repellent from vapour repellent (Bissinger & Roe 2013). Carroll *et al*. (2004) showed that DEET repelled *I. scpularis* and *A. americanum* but specific mention was not made as to whether the response was due to perception of the volatiles or by contact stimulus in the ticks. In contrast, McMahon *et al*. (2003) in their report, found no repellent activity of DEET on *Amblyomma variegatum* and *I. ricinus*, wherein they defined a repellent as ‘a compound whose vapour inhibits response to an attractant and a deterrent as a compound (independent of its vapour pressure) that inhibits the response to an arrestment stimulus’. Discriminating between repellency and deterrency is not always easy; however, results obtained in this study seem to suggest that the response elicited by garlic extracts on male and female ticks in the repellency bioassays is purely a result of deterrent stimulus.

In both repellency bioassays, initial kinesis, coupled with the natural tendency of ticks to climb (Carroll, Klun & Schmidtmann 1995; Carroll, Mills & Schmidtmann 1996), could have reduced response to the repellents. It is possible that the timespan of test period influenced the repellency. For example, relatively reduced repellency was observed in the Type B compared to the Type A bioassay with varying discriminating time periods of 45 s and 1 h, respectively. Furthermore, the interaction of males and females and members of the same sex during host seeking is not fully understood (Perritt, Couger & Barker 1993). In order to minimise any interactions between members of the different sexes, males and females were used separately in this study; however, generally, there were no significant differences in the responses of male and female ticks in this study. Aggregation behaviour was observed in the Type A repellency bioassay and it is possible that tick aggregation tendency could have interfered with tick response to the repellent in this study. On the other hand, releasing ticks in succession as was the case in the Type B repellency bioassay may have prevented natural interaction of ticks. Nonetheless, results obtained in the Type B bioassay give us an indication of the degree of avoidance of the extracts by the ticks and an indication of which sensory receptor could be responsible for the perception of stimulus. Naturally, one would think that as time progressed volatile compounds were lost to the environment, thus reducing the effectiveness of their extracts. According to Fradin and Day (2002), the complete protection time of most botanical repellents is short-lived (ranging from a mean of 3–20 min). It may be interesting to repeat the experiment after sufficient time was allowed for the volatile compounds to evaporate.

## Conclusion

DCM extracts of garlic repelled adults of *H. rufipes*. Discrepancies in results obtained in tick repellency bioassays suggest more than one *in vitro* bioassay may be needed to conclude with certainty that a plant extract is a tick repellent. Garlic bulb extract contains potential insect repellents such as thiophenes and dithiane, and further studies to evaluate the repellent effects of the individual compounds against ticks are recommended.
